# Building Confidence in Age-Friendly Care in Nursing Facilities and Care Systems through the Kansas 4M Partnerships

**DOI:** 10.1093/geroni/igaf122.3552

**Published:** 2025-12-31

**Authors:** Frances Yang, Shawna Wright, Barbara MacArthur, Tracy Davies, Jacqueline Beldeen, Colleen Paramesh, Kristine Williams, Teri Kennedy

**Affiliations:** University of Kansas, University of Kansas Medical Center, Kansas, United States; University of Kansas Medical Center, Kansas City, Kansas, United States; University of Kansas, Kansas City, Kansas, United States; Washburn University, Topeka, Kansas, United States; University of Kansas Medical Center, Kansas City, Kansas, United States; University of Kansas Medical Center, Kansas City, Kansas, United States; University of Kansas, Kansas City, Kansas, United States; University of Kansas Medical Center, Kansas City, Kansas, United States

## Abstract

Kansas 4M Geriatric Workforce Education Program is a statewide community-academic partnership to advance evidence-based Age-Friendly Health Systems (4Ms: What Matters, Medication, Mentation, and Mobility) care. In partnership between Kansas 4M, Project Extension for Community Healthcare Outcomes (ECHO), Kansas Nursing Workforce Center, and Advanced Nursing Education Workforce, the Kansas 4M Project ECHO series introduced and applied the 4Ms to the care of nursing home residents, such as communication strategies. This series was designed for Long-Term Services and Support (LTSS) staff, administration, nurses, nurse educators, advanced practice clinicians, physicians, and behavioral health clinicians (N = 37). Significant improvement in confidence was found after seven sessions for the statement: “I am confident in my ability to describe effective strategies to incorporate the 4Ms framework into areas of care for nursing facility residents and older adults such as chronic care management, end-of-life support, memory care, and telemedicine.” With responses ranging from Strongly Disagree (1 point) to Strongly Agree (5 points). Based on paired t-tests, the average increase was 1.59 points (Standard Deviation (SD)=1.12, t = 8.68, p < 0.001) in confidence from the beginning (Mean (M)=2.7, SD = 1.03) to the latest follow-up (M = 4.3, SD = 0.80). There was also improvement found for the statement: “I am confident in my ability to explain the importance of implementing age-friendly care in nursing facility and nursing care systems that support older adults” by an average of 1.38 points (SD = 1.09, t = 7.70, p < 0.001). The Kansas 4M model with Project ECHO has shown statistically significant evidence for improvement in confidence among health care workers in LTSS.

